# Screening for Type II L-Asparaginases: Lessons from the Genus *Halomonas*

**Published:** 2017

**Authors:** Zeinab Sharafi, Mahmood Barati, Mohammad Reza Khoshayand, Sina Adrangi

**Affiliations:** a *Department of Pharmaceutical Biotechnology, School of Pharmacy, Shahid Beheshti University of Medical Sciences, Tehran, Iran. *; b *Department of Drug and Food Control, Faculty of Pharmacy, Tehran University of Medical Sciences, Tehran, Iran.*

**Keywords:** Screening, L-asparaginase, *Halomonas*, Oligotrophy, Optimization

## Abstract

Among the two types of bacterial L-asparaginases, only type II enzymes have been used in the treatment of acute lymphoblastic leukemia owing to their higher affinity for L-asparagine. However, current screening media used for the isolation of L-asparaginase-producing microorganisms do not discriminate between the two types of L-asparaginase. During an optimization study conducted to increase L-asparaginase production by environmental *Halomonas* isolates, it was noticed that the pattern of L-asparaginase production in response to variations in glucose concentration varied between different isolates suggesting that they differ in their ability to produce type II L-asparaginases, an observation that was confirmed by further experiments. Bioinformatics analysis of available *Halomonas* whole genome sequences revealed that indeed some species of this genus possess both L-asparaginase types while others possess only type I enzymes. By comparing the growth pattern of these isolates on different media, we propose that by omitting glucose, reducing the concentration of L-asparagine and providing an alternative nitrogen source in L-asparaginase screening media it may be possible to differentiate between type I and type II activities.

## Introduction

Bacterial L-asparaginases (EC 3.5.1.1) have successfully been used in the treatment of acute lymphoblastic leukemia for decades. It is generally accepted that the major mechanism through which L-asparaginases exert their antileukemic effect is by depleting the extracellular L-asparagine pool and thus inhibiting the growth and proliferation of cancerous cells that, unlike normal cells, rely on exogenous asparagine for protein synthesis (1). Bacterial L-asparaginases are classified into two types (2, 3). Type I L-asparaginases (encoded by *ansA*) are homodimeric cytoplasmic enzymes that show low affinity to L-asparagine. On the other hand, type II L-asparaginases (encoded by *ansB*) are high affinity enzymes located in the periplasmic space that usually assume a homotetrameric configuration. The antitumor activity of L-asparaginases is related to their affinity for L-asparagine. The physiological concentration of L-asparagine in human blood is in the range of 40-80 μM that should be reduced to 0.1-0.2 μM in order for efficient leukemic cell death to occur (4). Only enzymes with a *K*_m_ around 10^-5^ M can exert such a strong effect (5). Typically, the *K*_m_ of type I L-asparaginases is in the millimolar range while that of type II L-asparaginases is two orders of magnitude lower (3). As a result, only type II enzymes have been used as therapeutic agents (2, 6-8). Despite the fact that only high-affinity L-asparaginases are of potential therapeutic value, current screening media used for the isolation of new L-asparaginase-producing bacteria not only do not differentiate between type I and II producers, but actually favor the detection of type I enzymes due to their composition as discussed later.

In a screening program conducted in our laboratories 5 *Halomonas* strains (designated H2, H3, H23, H27 and H28) with the ability to produce L-asparaginase were isolated from samples collected from several hypersaline environments (9). In preliminary purification studies, however, it was noticed that even with the isolate producing the highest amount of L-asparaginase (isolate H28; 1.8 U/mL) the produced L-asparaginase level was too low to provide sufficient quantities for biochemical and kinetics characterization. In an attempt to overcome this problem, statistical optimization studies were designed and conducted. While evaluating the effect of glucose on L-asparaginase production by these isolates, it was noticed that some isolates were able to produce both type I and type II L-asparaginases while others possessed only type I L-asparaginases. Using these isolates and taking into account the physiological functions of type I and II L-asparaginases, we developed a selection medium that can potentially be used for the isolation of type II L-asparaginase-producing microorganisms. These findings are reported in this paper.

## Experimental

*Culture conditions*

The basic production medium consisted of the following components (per liter): 20% glucose solution (10 mL), L-asparagine (5 g), KH_2_ PO_4_ (3 g), Na_2_HPO_4._2H_2_O (6 g), 1 M MgSO_4_.7H_2_O solution (1 mL), 0.1 M CaCl_2_.2H_2_O solution (1 mL) and NaCl (50 g) (10). The pH of the medium was adjusted to 7. Since our samples were collected from hypersaline environments the concentration of NaCl used in this medium is much higher than that of the original medium as described in reference 10.

**Figure 1:**
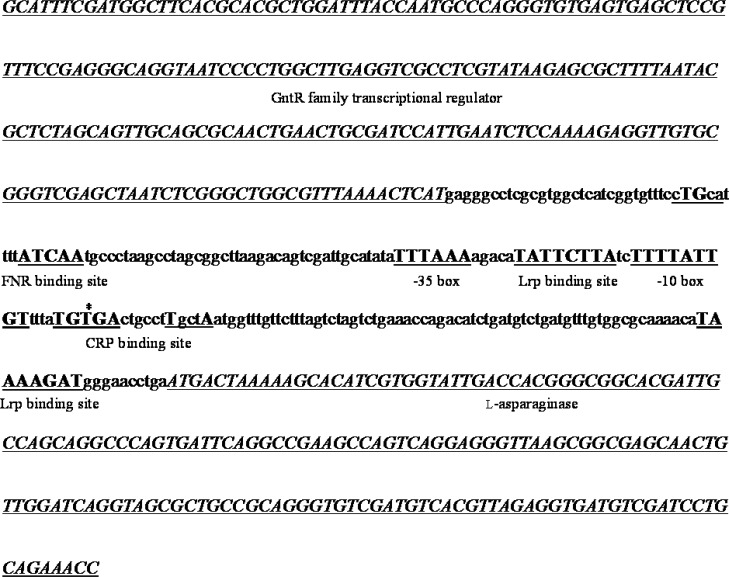
The regulatory region of the putative type II L-asparaginase gene of isolate H28. Genes are shown in italic, regulatory sequences in bold and all other regions in lowercase characters. The predicted transcription start site is marked with an asterisk

**Figure 2:**
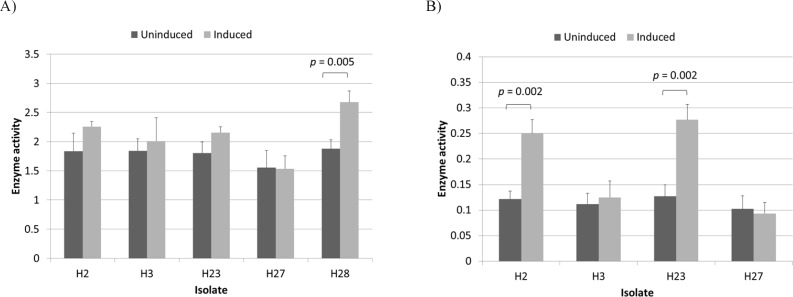
L-asparaginase activity of induced and uninduced *Halomonas* isolates measured at substrate concentrations of 10 mM (A) and 0.1 mM (B). Enzyme activity is given in U/mL/OD_600_. The *p*-values for significant differences (obtained through two-sample *t* test) are shown

**Table 1:** Growth of *Halomonas* isolates on different media. The basic selection medium was supplemented with the indicated carbon and nitrogen sources and growth was examined by visual inspection

**Medium**	**Carbon source**	**Nitrogen source**	**Growth**				
			**H2**	**H3**	**H23**	**H27**	**H28**
A	Glucose (4 mM)	NH_4_Cl (20 mM)	+	+	+	+	+
B	L-asparagine (90 mM)	Same as carbon source	+	+	+	+	+
C	L-asparagine (1 mM)	Same as carbon source	+	-	+	-	+
D	L-asparagine (0.1 mM)	Same as carbon source	-	-	-	-	-
E	L-asparagine (0.1 mM)	NH_4_Cl (20 mM)	+	-	+	-	+

A single colony of the *Halomonas* isolate was inoculated into 10 mL of Lysogeny broth (LB) medium with 1.5 M NaCl (10 g/L peptone, 5 g/L yeast extract and 87.6 g/L NaCl; all from Merck KGaA, Germany) and incubated overnight at 30 °C and 180 rpm. This preculture was used to inoculate 20 mL of the production medium in 100-mL Erlenmeyer flasks. These cultures were then incubated at 30 °C, 180 rpm for 24 h.

The basic selection medium was identical to the basic production medium except that glucose and L-asparagine were omitted. This medium was supplemented with various carbon and nitrogen sources as indicated in the text and solidified with 15 g/L agar (Liofilchem, Italy). 

*L* *-Asparaginase assay*

L-Asparaginase activity was measured using Nessler’s method (11). In brief, 100 μL of enzyme preparation was mixed with 400 μL of distilled water, 100 μL of 100 mM L-asparagine (Sigma-Aldrich, USA) solution and 500 μL of 50 mM Tris-HCl buffer (pH 8.6) and incubated at 37 °C for 30 min. The enzyme reaction was stopped by adding 50 μL of trichloroacetic acid (Sigma-Aldrich, Germany) (1.5 M). An aliquot of 200 μL of the above mixture was added to 2100 μL of distilled water and 250 μL of Nessler’s reagent (Sigma-Aldrich, Switzerland). The absorbance was measured at 436 nm. One enzyme unit was defined as the amount of enzyme that produces 1 μmole of ammonia in 1 min under assay conditions.

*Experimental design and statistical analysis*

The production medium was optimized in two steps. First, the most important factors affecting L-asparaginase production by *Halomonas* H28 and the range over which they were effective were determined. A total number of 11 parameters including the effect of all medium components (glucose, 2.5-20 g/L; L-asparagine, 2.5-20 g/L; KH_2_ PO_4_, 2.5-7.5 g/L; Na_2_HPO_4_, 2.5-7.5 g/L; 1 M MgSO_4_, 0.2-0.8 mL ; 0.1 M CaCl_2_, 0.1-0.4 mL; and NaCl, 5-150 g/L) plus the effect of replacing glucose with 2 alternative carbon sources (maltose, 2.5-15 g/L; and lactose, 2.5-15 g/L) and that of 2 physical factors (inoculum size, 1-15 %; and temperature, 27-36 °C) were studied individually in the indicated range (Table S1). In each case, all other parameters were kept constant. Results were compared through one-way ANOVA and Tukey’s HSD test using the IBM SPSS Statistics 22 software (IBM Corp., USA).

In the second steps, a central composite design was used to build a second-order model using Design Expert software version 7 (Stat-Ease Inc., USA). A total number of 20 experiments including 6 replicates at the central point were performed to investigate the effect of the three selected factors (L-asparagine, glucose and NaCl concentrations) in three levels each (Table S2). ANOVA was used to evaluate the effect of the variables and significant results were identified by a *p*-value of < 0.05. 

*Amplification and characterization of ansB regulatory region*

The regulatory region of the *ansB* gene from *H.* *huangheensis* (NZ_CP013106.1), *Halomonas* sp. KO116 (NZ_CP011052.1), and *Halomonas* sp. R57-5 (LN813019.1) were retrieved from the NCBI Nucleotide database. These sequences were aligned using CLUSTAL Omega and conserved regions were used to design primers ANSF (5´-TGCAGCATCTTGATGGCTTC-3´) and ANSR (5´-GGCGTTGCTGGGTTTCTG-3´) using Primer BLAST (12) (Figure S1). These primers were used to amplify a region of approximately 600 bp from each isolate using a peqSTAR thermocycler (PEQLAB Biotechnologie, Germany). The PCR program included an initial denaturation step at 95 °C for 300 sec, 30 cycles of denaturation at 95 °C for 30 sec, annealing at 60 °C for 30 sec, extension at 72 °C for 60 sec, and a final extension at 72 °C for 600 sec. Selected fragments were sequenced by a commercial company. The promoter regions of the obtained sequences were analyzed using BPROM (13), GeneMark (14) and NNPP (15) and the presence of carbon and fumarate response elements was investigated using ApE (http://biologylabs.utah.edu/jorgensen/wayned/ape/).

*Random amplified polymorphic DNA-PCR (RAPD-PCR)*

RAPD-PCR was performed using primers 5´-CCGCAGCCAA-3´ (P1) and 5´-GATGACCGCC-3´ (P2) in two separate reactions for each isolate (16, 17). The PCR program used for the first primer included 4 cycles of denaturation at 95 °C for 300 sec, annealing at 36 °C for 300 sec, extension at 72 °C for 300 sec followed by 30 cycles of denaturation at 95 °C for 60 sec, annealing at 36 °C for 60 sec, extension at 72 °C for 120 sec, and a final extension at 72 °C for 600 sec. The PCR program for the second primer was as follows: an initial denaturation step at 95 °C for 300 sec, 35 cycles of denaturation at 94 °C for 30 sec, annealing at 40 °C for 30 sec, extension at 72 °C for 120 sec, and a final extension at 72 °C for 600 sec.

*Type II* *L* *-asparaginase induction*

The induction medium was identical to the basic production medium except that glucose was omitted, L-asparaginase concentration was reduced to 0.1 mM and the medium was supplemented with NH_4_Cl as a nitrogen source at a final concentration of 20 mM. Cells were cultivated in either the optimized or LB medium with 1.5 M NaCl at 30 °C and 180 rpm for 24 h and harvested by centrifugation at 5000×g for 7 min. Cells were then washed twice using the induction medium and transferred to airtight sterile tubes containing induction medium without head space and incubated at 30 °C for 1h. It was later noticed, however, that incubation under normal conditions as described above in the induction medium was sufficient for induction. Controls were simply incubated for an additional hour under normal conditions without medium replacement. Results were compared using two-sample *t* test.

## Results and Discussion

Type I L-asparaginases are involved in nitrogen metabolism and appear to be expressed constitutively (2, 5 and 18). On the other hand, type II L-asparaginases appear to participate in carbon metabolism and their expression is tightly regulated by different factors (2). In *Escherichia coli* the expression of *ansB* is regulated by the action of two global transcription factors: the cAMP receptor protein (CRP) and the fumarate and nitrate reductase regulator (FNR) (5). The former regulates the expressions of genes involved in carbon source utilization under carbon-limited conditions while the latter is a regulator of genes for anaerobiosis (19, 20). In order to investigate whether the expression of type II L-asparaginase in *Halomonas* H28 is controlled by similar mechanisms, its regulatory region was sequenced and analyzed. Results are shown in Figure 1. An FNR binding site was found at position -83. This site is almost identical to that reported for the *H. Maura* *narGHJI* operon (21). It also appears to be in an appropriate distance from the predicted transcription start site (22). A CRP binding site that overlaps the predicted transcription start site was also identified. Although they are usually located at a position between -41and -82 relative to the transcription start site, examples of CRP binding sites overlapping the transcription start site have also been described in the literature (23). Finally, two leucine-responsive regulatory protein (Lrp) binding sites were found at positions -21 and +82. Lrp is another global transcription factor that regulates the expression of genes involved in amino acid transport, metabolism and utilization in *E. coli* (24). Thus, maximum Type II L-asparaginase expression in H28 is expected to occur under anaerobic conditions and in the absence of glucose. Since cultivation under these conditions results in very low cell densities, we attempted to maximize L-asparaginase production by first optimizing biomass production and then inducing enzyme production by transferring the resulting cells to the induction medium (25).

The PCR protocol used for the amplification of the type II L-asparaginase regulatory region in H28 was also successfully used to amplify the corresponding region in H23, however it failed to produce any bands in the anticipated size range with isolates H2, H3 and H27 (Figure S2). This was not surprising as the primers were designed using the three NCBI Genome entries whose 16S rDNA showed the highest level of similarity with that of H28 and were not meant to be universal. The promoter region of H23 was also sequenced and found to be almost identical to that of H28. This observation suggested that H23 and H28 may actually be two different isolates of the same strain. However, RAPD-PCR analysis revealed that these two isolates are closely related but not identical (Figure S3). Also, a comparison of the 16S rDNA sequences of these two isolates (9) showed that they share only 93.7% similarity (Figure S4).

After growth medium optimization and before induction (Figures S5 and S6), total L-asparaginase activity for isolate H28 in an optimized medium containing 14 g/L glucose, 12 g/L L-asparagine and 54 g/L NaCl reached 6.2 U/mL which is considerably higher than the activity observed in the original medium (1.8 U/mL). Since L-asparaginase activity per unit of OD600 remained constant for all measured points (p-value > 0.05) (Table S2 and Figure S6), it can be assumed that the observed increase in L-asparaginase activity resulted almost entirely from increased type I production as a result of increased cell density. It was then decided to induce type II L-asparaginase production to obtain sufficient amounts needed for purification and characterization studies which will be presented in another paper. Interestingly, during induction studies it was noticed that *Halomonas* isolates responded differently to the induction procedure. These differences were first observed in the optimized medium. Since this medium was optimized using only one isolate (H28), the experiments were repeated using LB medium with 1.5 M NaCl to eliminate the effect of any unknown factor that may influence the enzymatic properties of the isolates. The results are shown in Figure 2A. While L-asparaginase activity per unit of OD_600_ for H28 increased significantly after induction (*p*-value < 0.05), isolates H2, H3, H23 and H27 were apparently unaffected by the process. However, we speculated that since in some strains type II activity comprises only a small fraction of total L-asparaginase activity (6), it presence in these isolates may had been masked by variation in type I activity levels. In order to avoid this effect by reducing the contribution of type I activity, L-asparaginase activity was assayed at an L-asparagine concentration of 0.1 mM (5, 26). At this reduced substrate concentration a significant (*p*-value 

< 0.05) increase was observed with H2 and H23 but not with H3 and H27 (Figure 2B). These results indicated that isolates H2, H23 and H28 produce both type I and II enzymes while isolates H3 and H27 are capable of producing only type I L-asparaginases. It has already been shown that *Halomonas* is a heterogeneous genus that can be classified into two distinct subgroups based on 16S rDNA sequencing (27), however the presence of type II L-asparaginase does not appear to be related to this classification scheme as 16SrDNA sequence alignments revealed that H28 belongs to subgroup 1 while H2 and H23 belong to subgroup 2 (data not shown). An analysis of the eight complete *Halomonas* whole genome sequences available in the NCBI Nucleotide database revealed that four of them (NZ_CP013106.1, NZ_CP011052.1, NZ_CP019326.1 and NZ_CP019915) contained both type I and II L-asparaginases while the other four (NZ_CP007757.1, CP014226.1, FN869568 and NZ_CP020562.1) appeared to contain only type I enzymes. In all cases where a type II L-asparaginase gene was present (with the exception of NZ_CP019326.1 which is missing part of a contig at this location as indicated in the record), it was flanked by a number of genes that appear to be involved in the uptake and utilization of C_4_-dicarboxylates. Three of these genes encode the three different parts of a DctPQM family tripartite ATP-independent periplasmic (TRAP) transporter. TRAP transporters are a group of high affinity transporters that play an essential role in the uptake of different carbon an energy sources under oligotrophic conditions (28, 29). Among these transporters, the DctPQM family have specifically adapted to uptake C_4_-dicarboxylates such as fumarate and aspartate (30, 31). Considering the fact that aspartate is a major product of type II L-asparaginases, it is not surprising that the genes encoding the DctPQM systems in *Halomonas* species are organized into a cluster that includes the asparaginase gene. Interestingly, these clusters are all preceded by a GntR family transcriptional regulator gene oriented in the opposite direction; an arrangement reminiscent of a typical bacterial operon. As shown in Figure 1, the type II L-asparaginase gene of H28 is also preceded by a putative GntR regulator homolog. This combination (a substrate-specific transporter coupled with the corresponding cell-surface enzyme) is a feature used to adapt to different substrate concentrations by many aquatic bacteria (32).

All these data suggest that *Halomonas* type II L-asparaginases are involved in the provision of carbon under nutrient-poor conditions. If this is the case, only *Halomonas* isolates capable of producing type II L-asparaginases should be able to grow in minimal media containing low levels of L-asparagine as the sole source of carbon. In order to investigate this matter, we evaluated the growth of our isolates on a basic selection medium amended with different combinations of carbon and nitrogen sources (Table 1). It was notice that isolates H2, H23 and H28 were able to grow on media containing low millimolar and, provided that the medium is supplemented with sufficient amounts of an appropriate nitrogen source, even sub-millimolar concentrations of L-asparagine as the sole source of carbon. However, even after several days of incubation bacterial growth was minimal and barely visible to the unaided eye. This was not unexpected as slow growth is one of the two main strategies used by microorganisms to strive in nutrient-deficient environments (33, 34). The second strategy is known as the mixed substrate growth strategy and its potential implications are discussed in the next section.

These results call into question the suitability of currently used L-asparaginase screening media for isolating type II L-asparaginase-producing microorganisms. The original medium used to isolate our study strains is described in reference 10. It is essentially a screening medium based on the M9 minimal medium in which the pH changes resulting from L-asparaginase activity is visualized using phenol red. The only nitrogen source provided in this medium is L-asparagine and it contains relatively high concentrations of glucose (11 mM). Most obligate oligotrophs would not be able to grow on such carbon-rich media unless they were subcultured in an appropriate adaptation medium for several generations (35). As for facultative oligotrophs, although they would probably form visible colonies on this medium, it is very unlikely that they would produce detectable levels of type II L-asparaginases in the presence of such high levels of glucose. These microorganisms would most likely go unnoticed unless they also possessed type I L-asparaginases. As stated above, all our type II-producing isolates are able to produce type I L-asparaginases as well, which explains why we were able to isolate them using this medium. This is not necessarily always the case. For example, the complete genome sequence (NCBI Genome accession CP0195450) of the newly identified *Halioglobus japonicus* (36) appears to contain two type II but no type I L-asparaginases. Thus, selection media containing low levels of L-asparagine as a sole source of carbon and sufficient amounts of a suitable nitrogen source (*e.g.* media C and E) may provide a better alternative for isolating type II L-asparaginase producers.

*Limitations and Possible Solutions*

It should be noted that the composition of the media introduced in this paper needs to be adjusted according to the requirements of the species under study. For example, sodium ions have been shown to suppress the production of type II L-asparaginases in some bacterial strains (37). Using potassium salts may alleviate the problem in such cases. In addition, for samples collected from normal habitats the concentration of NaCl should be reduced to its original level described in reference 10. Growth media used for the isolation of bacteria from environmental samples also usually need to be supplemented with vitamin and trace element solutions (38-40). The concentration of L-asparagine is another important factor that requires careful adjustment. Sub-millimolar L-asparagine concentrations were successfully used to select type II L-asparaginase-producing isolates in this study (medium E). Similarly, it has been shown that some *Halomonas* strains are able to grow on different media containing sub-millimolar (40) and even micromolar (41) concentrations of a single or a combination of appropriate carbon sources. It should be remembered, however, that the ability to grow in nutrient-poor environments depends on several factors including the presence of high affinity transport systems (34, 42). Both the number (43) and the affinity (32) of these transporters affect the host’s ability to grow on such media. In addition, some microorganisms use the mixed substrate growth strategy at sub-millimolar concentrations (33) and thus may not be able to grow on a single substrate despite possessing the necessary enzymes. Increasing the concentration of L-asparagine to low millimolar levels (medium C) may increase the chance of isolating such microorganisms, but it may do so at the cost of compromising selectivity as alternative pathways might be activated at higher concentrations. For example, the low-affinity L-asparagine uptake systems (the products of the *ansP* genes) of *E. coli* and *Salmonella enterica* are activated at L-asparagine concentrations above 0.1-1 mM and are capable of increasing the intracellular concentration of L-asparagine to sufficiently high levels to be utilized as substrate by type I L-asparaginases (2, 18 and 26). This phenomenon may explain why all our isolates were able to grow on media containing high millimolar L-asparagine concentrations (medium B). Also, some bacteria such as *E. coli* DH5α seem to produce type II L-asparaginase only under anaerobic conditions (8) while in others anaerobiosis has a negative effect (37). Even if all these considerations are taken into account, this method still suffers from a major drawback, *i.e*., the extremely slow growth of a considerable proportion of oligotrophic bacteria on agar plates that may necessitate very long incubation times (43, 44). This problem can be avoided by using methods based on extinction dilution combined with fluorescence microscopy (38, 45). These methods are more expensive and laborious but are capable of attaining cultivation efficiencies of approximately 15%. Nevertheless, it has been shown that solid carbon-limited media supplemented with millimolar concentrations of ammonium ions can reliably be used for the isolation of oligotrophs from aquatic habitats (44) which is in accordance with the results presented in this paper.
